# Groupthink: chromosomal clustering during transcriptional memory

**DOI:** 10.15698/mic2015.12.244

**Published:** 2015-11-26

**Authors:** Kevin A. Morano

**Affiliations:** 1 Department of Microbiology and Molecular Genetics, University of Texas Medical School at Houston, Houston, TX 77030 USA.

**Keywords:** transcriptional memory, chromatin, nuclear pore complex, DNA zip code

Gene expression is regulated by the dynamic nature of DNA modification, histone modification, and recruitment of activating and repressing protein complexes at specific loci. Chromatin itself is further organized structurally into regions of varying accessibility, an aspect of which includes transient or sustained redistribution of portions of the genome from the nucleoplasm to the nuclear periphery. Targeting of genes to the nuclear membrane, frequently via association with components of the nuclear pore complex (NPC), has both positive and negative effects on the timing and magnitude of gene expression 1. Moreover, chromatin localization to the nuclear membrane endows certain genes with a form of multigenerational transcriptional memory after inactivation, allowing faster reactivation in the progeny upon return to inducing conditions 2.

The mechanisms underlying these various phenomena are beginning to be understood at the molecular level, perhaps best exemplified by studies in the baker’s yeast *Saccharomyces cerevisiae*, where precise manipulation of environmental conditions can be coupled with state of the art single-cell imaging and genetics. For example, genes have been found to localize to the nuclear periphery in response to nutrient shift (*GAL1-10, INO1, SUC2, HXK1*) and heat shock (*HSP104, TSA2*) (summarized in ref 3). This dynamic association requires NPC proteins and, in the case of *INO1*, two sequence elements (gene recruitment sequence I and II; GRS) in the promoter 4. While the role of GRSII remains unclear, GRSI recruits the Put3 transcription factor as an intermediary for NPC targeting 5. After shift to repressing conditions, the *INO1* locus remains associated with the nuclear periphery for multiple generations, conferring transcriptional memory via stabilization of a poised RNA polymerase II complex 6,7. This phenomenon requires a different promoter element termed the memory recruitment sequence (MRS) and a different nuclear pore protein (Nup100) than those involved in GRS recruitment 6. Therefore, unique “DNA zip codes” dictate targeting of a gene to the nuclear periphery via distinct protein interactions. Importantly, these targeting events are independent of each other - GRSI, II and Put3 are not required during memory and the MRS is apparently dispensable for localization during gene activation 6.

The Brickner laboratory has paved the way in our understanding of these events, and elucidated much of the molecular detail. Central to their discoveries has been the development of methods to quantitatively assess sub-nuclear chromatin positioning. These approaches utilize DNA arrays consisting of Lac (LacO) and Tet (TetO) Operator elements integrated at loci of interest paired with fluorescent protein-DNA binding protein fusions. Cells bearing these endogenous and/or ectopic constructs can then be visualized with confocal microscopy and intra-nuclear distances measured (i.e., gene-gene or gene-nuclear periphery) for individual cells within a population. A key observation derived from these analyses was that two copies of the same locus, for example *INO1/INO1* in a diploid cell, identified with different color fluorophores, were found to cluster together in three-dimensional space 8. Moreover, two independent loci, each bearing a GRSI element, likewise cluster during activation 8.

In the article published in this issue of *Microbial Cell*, Brickner and co-workers reveal that clustering also occurs during transcriptional memory 9. Multiple aspects of *INO1* memory clustering differentiate this phenomenon from that observed upon gene activation. Transcriptional memory at the *GAL1-10* locus requires the nuclear basket protein Mlp1, in contrast to *INO1* which targets to the NPC through Nup100 6,10. Consistently, *GAL1* and *INO1*, when differentially labeled, were found not to cluster after shift to repressing conditions, indicating specificity 9. The MRS zip code was also shown to be required for clustering, functionally linking this feature with targeting to the nuclear periphery during memory. Fascinatingly, the MRS was seen in this study to be insufficient for memory-based clustering - both GRSI and GRSII elements are also required. The same held true for the role of the GRSI binding protein Put3 and the gene activation nuclear periphery recruitment protein Nup100 9. Therefore, clustering during transcriptional memory requires previous clustering during gene activation, and each process relies on its own previously described components.

One of the less well understood features of gene localization at the nuclear periphery is that localization is dynamic during the cell cycle. Stress-activated genes such as *INO1, GAL1* and *HSP104* transit back into the nucleoplasm (as defined by average locus-periphery distances) during S phase, only to re-localize at the periphery before mitosis 11. Strikingly, gene clustering is maintained during this period, formally distinguishing clustering from NPC/nuclear membrane localization. In contrast, genes localized at the nuclear periphery during memory remain throughout the cell cycle 7. When Brickner *et al.* categorized clustering with respect to bud stage, they found that while un-budded or small-budded cells (G1/S) maintained memory-based clustering, large-budded cells (G2/M) did not, even though both *INO1* loci remained associated with the nuclear periphery 9. These events are therefore uncoupled. It is additionally interesting that despite undergoing closed mitosis (the nuclear membrane in budding yeast does not break down during mitosis and reform, as it does in most other eukaryotes 12), chromatin association with the membrane is temporarily altered. This is in contrast to spindle pole bodies, which remain intact and inserted into the nuclear membrane, anchoring microtubules throughout the cell cycle.

**Figure 1:**
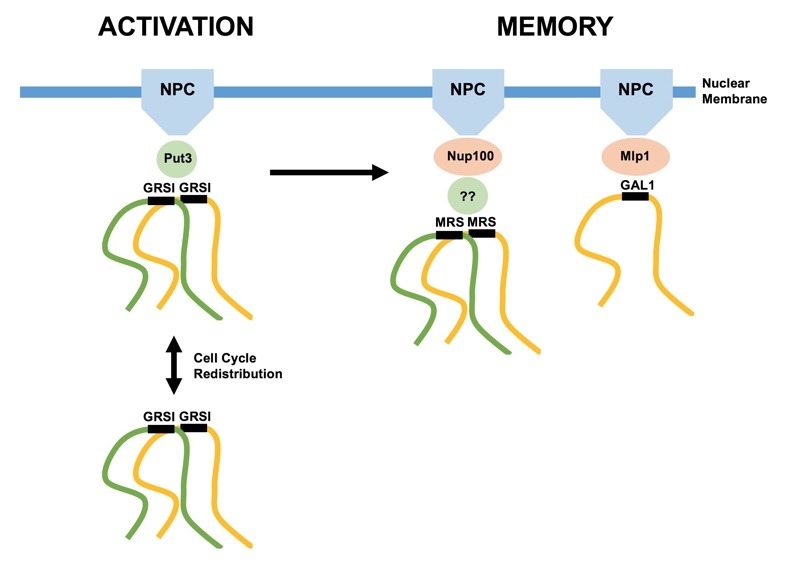
FIGURE 1: MRS-dependent interchromosomal clustering during epigenetic transcriptional memory. Portions of homologous chromosomes (yellow, green) are depicted clustering via their respective DNA zip code elements, associating with intermediary proteins and nuclear pore complexes (NPC). GRSI-mediated clustering is retained throughout the cell cycle independently of nuclear membrane binding. Memory-based clustering is specific to given genes/zip codes, possibly by virtue of distinct NPC adapter proteins. Memory-based clustering is dependent on prior clustering via GRSI elements during the activation phase.

This work further illuminates the complex and dynamic architecture of control of gene expression by chromatin localization, but raises even more interesting questions as it does so. Chief among these is the role of clustering with respect to targeting to the nuclear periphery. Because loci with the same DNA zip code will both cluster and target to the periphery, they appear to be localizing to the same location - possibly a specific NPC. However, clustering is retained after disengagement from the membrane, suggesting that it is not the targeted location that is specific but rather the association of different portions of chromatin. What is the fate of the nuclear pore proteins involved in nuclear membrane targeting? Do they release from the NPC with the chromatin, or release from the chromatin and remain with the NPC? The fact that clustering during gene activation is a prerequisite for clustering during transcriptional memory, while utilizing distinct DNA elements (and by extension, distinct DNA binding proteins) implicates a functional transfer of chromatin oversight from one system to another. It also suggests that the memory system is incapable of initiating clustering through the MRS zip code on its own. Identification of the as yet unknown MRS-binding protein (in addition to Nup100 within the NPC) may shed light on the molecular mechanisms that promote memory, defined by the rapid reinstatement of active transcription at a locus. This is thought to be achieved by stabilization of a pre-initiation transcriptional complex and a poised polymerase at the promoter, events that may be facilitated by insertion of the histone variant H2A.Z into flanking nucleosomes 6,13. It is not difficult to imagine a scenario where these events are coordinated via chromatin clustering, and perhaps accelerated via association with NPC, thus increasing local concentration of key components.

As with other epigenetic regulatory mechanisms, clustering and sub-nuclear localization of specific loci allow transient or sustained modulation of gene expression in the absence of permanent nucleotide changes in the genome. Such layers of control provide an attractive target for adaptive selection - organisms capable of re-activating stress response genes faster than their neighbors are likely to gain competitive advantage, without the possible trade-offs that arise with spontaneous mutation. Moreover, the trans-generational nature of transcriptional memory satisfies the tenet that selection can only act on heritable traits. Thus, cellular memory becomes organismal memory. Unraveling the complex language of control of gene expression via sub-nuclear positioning in yeast and other model systems such as Drosophila may have broader impacts to human health.

Potentially, thousands of human genes exhibit association with nuclear pore proteins 14,15. Human Nup98 promotes expression of developmental genes in neuronal precursor and stem cells, has been linked to transcriptional memory, and is even associated with several acute myeloid leukemias due to a chromosomal translocation fusing Nup98 with the DNA binding domain of HOXA9 16. Histone methylation at promoters during memory is also regulated in a manner that requires Nup98 in HeLa cells, and its homolog Nup100 in yeast 10,15. As was the case with chromatin modification a decade ago, we have begun to learn the words to a new spatial positioning code. We must now learn the syntax.
